# Pharmacological and computational evaluation of Sapodilla and its constituents for therapeutic potential in hyperactive gastrointestinal disorders

**DOI:** 10.22038/IJBMS.2019.35595.8488

**Published:** 2020-02

**Authors:** Muhammad Bilal Riaz, Arif-ullah Khan, Neelam Gul Qazi

**Affiliations:** 1 Riphah Institute of Pharmaceutical Sciences, Riphah International University, Islamabad

**Keywords:** Anti-diarrheal, Anti-secretory, Anti-spasmodic, Anti-ulcer, Manilkara zapota, Molecular docking

## Abstract

**Objective(s):**

This study was designed to investigate various gastrointestinal effects of *Manilkara zapota* (Sapodilla), exploring its anti-diarrheal, anti-secretary, anti-spasmodic, anti-ulcer and anti-motility potential.

**Materials and Methods:**

Antidiarrheal and anti-secretary activities were investigated using castor oil induced diarrhea and castor oil induced fluid accumulation. Isolated rabbit jejunum tissues (antispasmodic) were employed for *in vitro* experiments. Antiulcer, antimotility and molecular docking were performed using ethanol-HCl induced ulcer assay, charcoal meal transit time and Auto Doc Vina.

**Results:**

Mz.Cr exhibited protection against castor oil-induced diarrhea (*P<*0.05 vs. saline group) and dose-dependently inhibited intestinal fluid secretions (*P<*0.001 vs. castor oil group). Mz.Cr caused relaxation of spontaneous and K^+^ (80 Mm)-induced contractions with EC_50_ values of 0.11mg/ml (0.08-0.1, n=4) and 0.16 mg/ml (0.09-0.2, n=4) respectively (^*^P<0.05^**^P<0.01 ^***^P<0.001). It showed protective effect against gastric ulcers induced by ethanol-HCl (*P<*0.001 vs. saline group). Mz.Cr reduced distance travelled by charcoal meal (*P<*0.001 vs. saline group). Plant constituents: caffeoylquinic acid and methyl 4-O-galloylchlorogenate showed high binding affinities (E-value≥-6.5 Kcal/mol) against histaminergic H_2_ receptors, H^+^/K^+^ ATPase pump and voltage gated L-type calcium channels, while possesses moderate affinities (E-value≥8 Kcal/mol) against histaminergic H_1_, muscarinic M_1_, M_3_ and mu-opioid, whereas lower affinities (E-value≥9.5 Kcal/mol) vs. calmodulin, adrenergic α_1_, phosphodiesterase enzyme and dopaminergic D_2_ receptors. Lupeol-3-acetate and β-amyrin-3-(3’-dimethyl) butyrate observed weak affinities.

**Conclusion:**

In present study, *M. zapota* is reported to exhibits anti-diarrheal, anti-secretory, anti-spasmodic, anti-motility, anti-ulcer effects and computational binding affinities against gastrointestinal targets.

## Introduction

Gastrointestinal ailments are very common among the people of Asia and medical practitioners believe that it is a root cause for the occurrence of several other co-morbidities. Modern day medicine has so far does not produced any efficacious remedial drug against gastrointestinal disorders. It only gives temporary relief but with side effects. However, traditional herbal medicines have got excellent economical and long lasting potential to treat digestive system disorders (1). These natural products have been a significant source and major contributor to the present day commercial medicines and several drug lead molecules. About 61% of drugs introduced worldwide are derived from natural products (2). Screening of crude plant extracts ease the way for discovery of novel bioactive compounds and their structure elucidation can open the window for new synthetic preparations. For particular therapeutic purposes, pure bioactive compounds can be made in suitable dosage form and their accurate doses can be find out (4). Edible fruits being potential sources of functional foods and its phytoconstituents often serves the purpose in treating and curing several chronic diseases. Use of edible fruit extracts have been reported by several researchers for their gastrointestinal activities (5). 

*Manilkara zapota *L. commonly known as “Sapodilla” and locally “Chiku” belongs to the family of Sapotaceae and is an evergreen, depilated tree up to 15 m in height. Asia is a major cultivator of this species, though it is native to Mexico and Central America (6). *M. zapota *has been used traditionally in fever, hemorrhage, wound healing, ulcer, arthritis, pulmonary diseases, rheumatism, and as antifungal agent (7). Its use as laxative and for treating constipation and diarrhea, further enhance its ethnomedicinal importance. Fruits are used in traditional medicines as anti-oxidant, due to their polyphenolic content (8).

*M. zapota *is reported with presence of polyphenolic compounds like tannins and flavonoids (9). Also, triterpenes were previously isolated from these fruits. Its methanolic extracts contain dihydromyricetin, quercitrin, myricitrin, catechins and gallic acid (7). Recently some novel triterpenes have been identified as 4-caffeoylquinic acid (cryptochlorogenic acid), lupeol-3-acetate, methyl 4-O-galloylchlorogenate and β - amyrin-3-(3’-dimethyl) butyrate (10).

**Figure 1:**
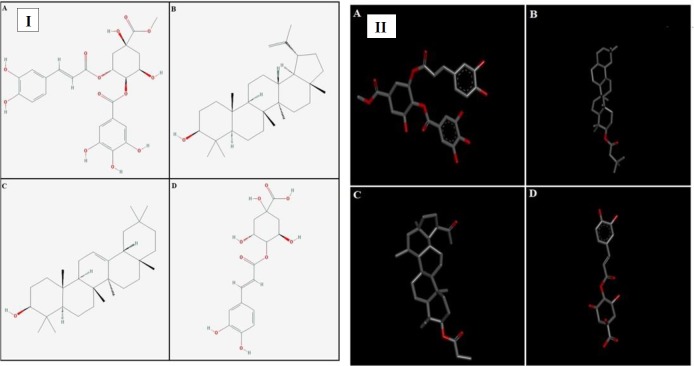
Panels [I] and [II] represents 2D and 3D structures of (A) methyl 4-O-galloylchlorogenate, (B) β-amyrin-3-(3’-dimethyl) butyrate, (C) lupeol-3-acetate and (D) 4-caffeoylquinic acid respectively

In the present study, we report anti-diarrheal, anti-secretary, anti-spasmodic, anti-motility and anti-ulcer effects. Extensive folkloric uses and previous studies were used as a baseline data to validate aforementioned ethnomedicinal uses of the plant. Molecular docking of its constituents with known structure is done to find out the potential lead molecule responsible for pharmacological effects. The 2D and 3D structures of the plant constituents: 4-caffeoylquinic acid (cryptochlorogenic acid), lupeol-3-acetate, methyl 4-O-galloylchlorogenate and β - amyrin-3-(3’-dimethyl) butyrate are presented in Figure 1.

## Materials and Methods

***Experimental procedures***

Superior quality of *M. zapota *fruit weighing 4 kg was purchased from local market in Feb 2017. Plant was authenticated by a taxonomist Dr. Mushtaq Ahmad, at Department of Plant Sciences, Quaid-a-Azam University, Islamabad. Voucher specimen no. (ISL-B-23) was collected after submitting sample of specimen of these species to the herbarium at same department. The fruit (4 kg) was air-dried, crushed into powdered form and extracted at room temperature with aqueous-methanol (70:30) three times to obtain *M. zapota *crude extract (Mz.Cr).

***Chemicals***

Atropine sulphate, omeprazole, verapamil, loperamide, papaverine, acetylcholine, charcoal, methanol and ethanol (Sigma Chemicals Co, St Louis, MO, USA) were used. Castor oil was obtained from KCL Pharma, Karachi, Pakistan.

***Experimental animals and housing conditions***

Sprague-Dawley rats (180-220 g), BALB/c mice (25-30 g) and rabbits (1.0-1.2 kg), of either sex were obtained from animal house of the Riphah Institute of Pharmaceutical Sciences (RIPS) Islamabad. The animals were kept in plastic cages at standard temperature (23-25 ^°^C). They were fed with standard animal feed and tap water *ad libitum*. Animals were fasted before each experiment for 24 hr. All the animal experimental protocols were approved by Research and Ethics Committee of RIPS (Ref. No. REC/RIPS/2017/008) which were performed in accordance with the guidelines of “Principles of Laboratory Animal care” (11).

***Phytochemical analysis***

Detection of major secondary metabolites presence such as glycosides, anthraquinones, steroids, flavonoids and tannins was carried out in Mz.Cr according to standard procedure (12) with slight modifications. 

***Castor oil induced diarrhea***

 Previously reported method was used for this study (13). All the test animals were fasted for 24 hr prior to commencement of experimentation. The floor of cage was lined with blotting paper in which animals were placed. First group was assigned as negative control group and received normal saline (10 ml/kg) orally, while second group was given with a dose of loperamide hydrochloride (10 mg/kg, p.o.) and assigned as positive control. Third, fourth and fifth groups received 50, 100 and 300 mg/kg body weight of the extract orally respectively. One hr after administration of the respective doses and treatments, all animals received (10 ml/kg, p.o.) of castor oil. Post treatment evaluation was carried out after waiting 4 hr in order to analyze the diarrheal droppings presence, absence of diarrheal droppings was documented as a positive result. Results were analyzed by applying Chi square test.

***Assessment of intestinal fluid accumulation***

Intestinal fluid accumulation was determined using the method as described previously (14). To study the intestinal fluid accumulation, entero-pooling assay was used. Overnight fasted mice were taken and put into five assigned cages with five mice in each. Group I and II were administered normal saline (10 ml/kg) and castor oil (10 ml/kg, p.o.) respectively. Extract doses of 50, 100 and 300 mg/kg intraperitoneally were given to Group III, IV and V respectively. Standard drug atropine at dose 10 mg/kg was given to last group, 1 hr prior induction with castor oil (10 ml/kg, p.o.). Mice were sacrificed after 30 min, then intestine was removed and weighed. The results were articulated as: (Pi/Pm) x 1000 where, Pi is the weight (g) of the intestine and Pm is the weight (g) of the animal. 

***Isolated tissue preparation***

Rabbits fasted for 24 hr before experiment but they had a free access to water. Jejunal portion was isolated after cervical dislocation of rabbit and washed with Tyrode’s solution. Jejunal segment of 2 cm length was suspended in tissue bath containing Tyrode’s solution. Temperature of bath was kept at (37 ^°^C) and proper aeration of 95% O_2_ and 5% CO_2_ (carbogen) is ensured. An initial load of 1 g was applied to each tissue and was allowed to equilibrate for 30 min before the addition of any drug. Following equilibration period, each preparation was then stabilized with sub-maximal concentration of ACh (0.3 μM) at 3 min interval until constant responses were recorded via a force displacement transducer (model FT-03) coupled with bridge amplifier and power Lab 4/25 data acquisition system connected to computer running Lab-Chart 6 software (AD Instrument, Sydney Australia). The effects of Mz.Cr at doses (0.01-3mg/mL) was recorded as the % change in the voluntary contractions of jejunum (15).

***Ethanol-HCl induced ulcer assay***

Rats weighing 250-280 g of either sex were distributed in 5 groups (n=5). Group 1 served as a negative control received normal saline 10 ml/kg body weight, group 2 received 20 mg/kg, (p.o.) omeprazole as standard drug; group 3, 4 and 5 received 50, 100 and 300 mg/kg, (p.o.) of Mz.Cr respectively. All the animals were treated with 1 ml/100 g of ethanol-HCl mixture (p.o.) i.e. (0.3 M Hydrochloric acid and ethanol 60%) after 1 hr to induce gastric ulcer. Animals were sacrificed via cervical dislocation 1 hr after administration of ethanol-HCl mixture. The stomachs were removed and lesion index was estimated by measuring each lesion in mm along its greater curvature. Surface area of each lesion was measured and scoring was done as described previously (16). For each stomach lesion, ulcer index was taken as mean ulcer score (US) such as; (0: no ulcer, 1: US≤0.5 mm^2^, 2: 0.5<US≤2.5 mm^2^, 3: 2.5 mm^2^<US≤5 mm^2^, 4: 5 mm^2^<US ≤10 mm^2^, 5: 10 mm^2^<US ≤15 mm^2^, 6: 15 mm^2^<US≤20 mm^2^, 7: 20 mm^2^<US≤25 mm^2^, 8: 25 mm^2^<US≤30 mm^2^, 9: 30 mm^2^<US≤35 mm^2^ and 10: US>35 mm^2^). For each stomach injury sum of the lengths (mm) of all sores was utilized as the ulcer index (UI). The gastro protective assessment was displayed as an inhibition percentage (I%) calculated by the following formula:

I (%) = (USc-USt) 100/USc

Where USc=ulcer surface area of control and USt=ulcer surface area of test drug group.

***Charcoal meal transit time***

Gastrointestinal transit time was estimated utilizing the charcoal meal test (17). Rats were fasted for 24 hr, the test groups received the extracts at 50, 100 and 300 mg/kg body weight doses, where as positive control group received atropine sulfate (0.1 mg/kg, IP), while the negative control group received normal saline (10 ml/kg, p.o.). 30 mins after all treatments, all the animals were sacriﬁced. The small intestine was excised after which the distance travelled by charcoal meal through the organ was expressed as a percentage of the length of the small intestine according to the following expression.

Intestinal transit(%)=(Distance moved by charcoal meal/ total length of intestine) (cm)×100.

***Acute toxicity***

Mice were divided in 3 groups of 5 mice each. The test was performed using increasing doses of the plant extract (3 and 5 g/kg) given in 10 ml/kg volume. Saline (10 ml/kg, p.o, negative control) was administered to one group. Twenty-four hr post study the mice were observed for mortality (18).

***Computational studies***

**Figure 2:**
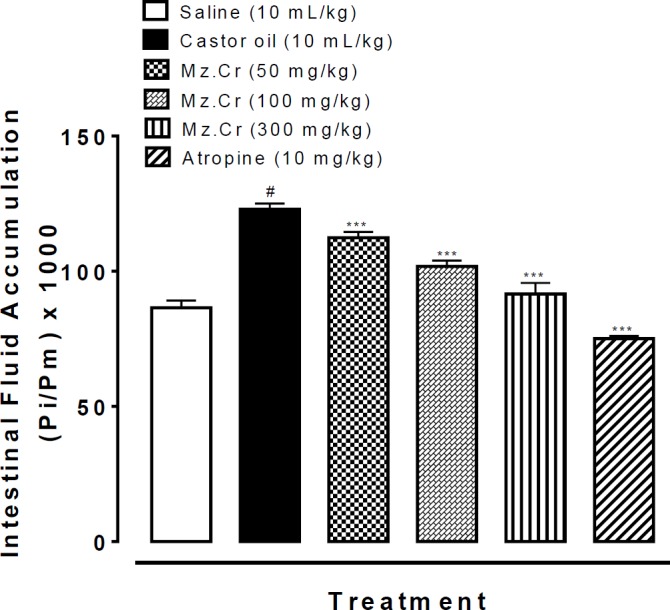
Effect of *Manilkara zapota* crude extract (Mz.Cr) and atropine on castor oil induced fluid accumulation in mice. Results are expressed as mean±SEM, n=5. Anti-secretory effect is expressed as Pi/Pm x 1000 (g) where Pi is the weight of the small intestine and Pm is the weight of mouse; ^#^*P<*0.001 vs. saline group, ^***^P<0.001 vs. castor oil group, one-way analysis of variance with *post hoc* Tukey’s test

**Figure 3:**
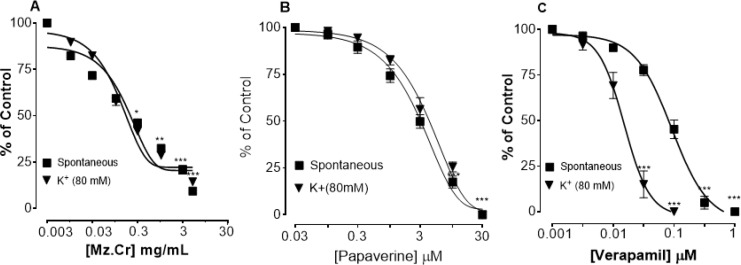
Dose-dependent inhibitory effect on spontaneous and K^+ ^(80 mM) induced contractions of (A) *Manilkara zapota* crude extract (Mz.Cr), (B) papaverine and (C) verapamil in isolated tissue preparations. *P<0.05 **P<0.01 ***P<0.001 one-way analysis of variance with *post hoc* Tukey’s test. Result expressed as mean±SEM, n=3-5

**Figure 4:**
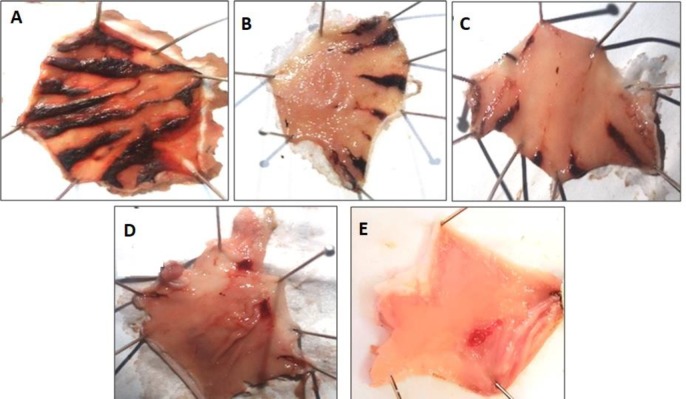
Gross-appearance of gastric mucosa in rat: (A) pretreated with saline, 10 ml/kg (ulcer control). Severe injuries are seen, as ethanol-HCl (1 ml/100 g) produced excessive hemorrhagic necrosis of gastric-mucosa (B, C and D) pretreated with *Manilkara zapota* crude extract (Mz.Cr) at doses of 50, 100, 300 mg/kg and (E) pretreated with omeprazole 20 mg/kg. The injuries reduce with increase of Mz.Cr doses and omeprazole compare with ulcer-control. At 300 mg/kg, Mz.Cr showed most efficacious gastro protective action

3-D structures of the test compounds (β-amyrin-3-(3’-dimethyl) butyrate, methyl 4-O-galloylchlorogenate, 4-caffeoylquinic acid and lupeol-3-acetate) were constructed by using the software of Gauss View 5.0 (Figure 2). Three dimensional structures of reference drugs were prepared through Discovery Studio Visualizer (2016) as shown in Figure 3. Reference drugs included phenoxy benzamine, verapamil, calmidazolium, domperidone, ranitidine, pirenzapine, atropine, loperamide, omeprazole, papaverine and pyrilamine. 3-D structures of selected targets possibly involved in the gut physiology, were retrieved from the website of RCSB protein data bank as represented in Figure 4. Selected targets included adrenergic α_1_ receptor (PDB ID:35348), muscarinic M_1_ (PDB ID:5CXV), muscarinic M_3_ (PDB ID: 4U14), dopaminergic D_2_ (PDB ID: 6CM4), calmodulin (PDB ID: 1CTR), mu-opioid (PDB ID: 5C1M), voltage gated L-type calcium channel (PDB ID: 1T3S), histaminergic H_1_ (PDB ID: 3RZE), histaminergic H_2_ (PDB ID: P25021), H^+^/K^+^ ATPase (PDB ID: 5YLU) and phosphodiestarase enzyme (PDB ID: 3G4K). Autodock Vina which is a geometry based automatic docking tool is used through which molecular docking was performed. Evaluation of docking results was based on atomic energy in Kcal/mol (19). Assessment in 2-D design was made to check the most extreme restricting interactions of complex framed amongst amino acid residues and ligands including: valine (VAL), alanine (ALA), proline (PRO), arginine (ARG), lysine (LYS), glycine (GLY), glutamine (GLN), asparagine (ASN), cysteine (CYS), methionine (MET), glutamic acid (GLU), histidine (HIS), phenylalanine (PHE), isoleucine (ILE), tyrosine (TYR), serine (SER), threonine (THR), aspartic acid (ASP) and tryptophan (TRP).

***Statistical analysis***

Data was expressed as mean±SEM (n=5) and median effective concentrations (EC_50_) having 95% confidence intervals. Statistical analysis of the results were analyzed using one-way ANOVA followed by *post hoc* Tukey’s test. Chi square test was used in the case of the anti-diarrheal data, where *P*<0.05 was regarded as significant. Non-linear regression using Graph Pad program (GraphPAD, SanDiego, CA-USA) was used to analyze the concentration-response curves.

## Results

***Phytochemical profile***

Qualitative phytochemical analysis of Mz.Cr showed the presence of flavonoid, phenols, triterpenes, lignin, unsaturated sterols and carbohydrates. 

***Effect of Mz.Cr on castor-oil induced diarrhea***

Mz.Cr exhibited a dose-dependent (50-300 mg/kg) protective effect against castor oil-induced diarrhea in mice. The negative control group (saline treated) did not show any protection against castor oil-induced diarrhea. Pretreatment of animals with the Mz.Cr, showed 20% protection from diarrhea at 50, 40% at 100 and 80% protection at 300 mg/kg (*P*<0.05 vs. saline group). Loperamide (10 mg/kg) showed 100% protection from diarrhea (*P*<0.01 vs. saline group) in the positive control group (Table 1). 

***Effect of Mz.Cr on intestinal fluid accumulation***

**Figure 5:**
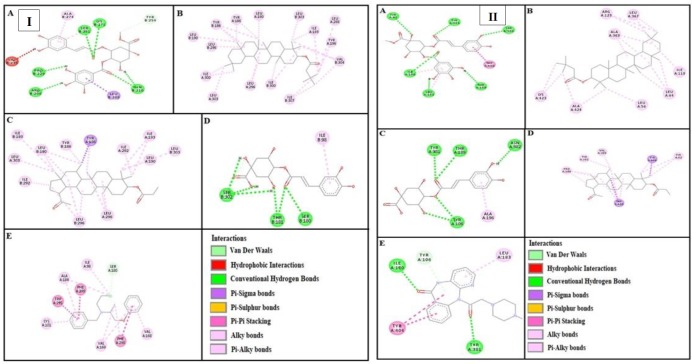
Panels [I] and [II] shows (A), (B), (C) and (D) interactions of methyl 4-O-galloylchlorogenate, β-amyrin-3-(3’-dimethyl) butyrate, lupeol-3-acetate and 4-caffeoylquinic acid against targets: adrenergic α1 and muscranic M1 receptors respectively. (E) represents phenoxy benzamine and pirenzepine interactions

When tested against castor oil-induced intestinal fluid accumulation in mice, Mz.Cr exhibited a dose-dependent (50-300 mg/kg) anti-secretory effect. Intestinal fluid accumulation in the saline treated group was 81.9±0.84 (mean±SEM, n=5), whereas in the castor oil-treated group it was 122.5±0.55 (*P*<0.001 vs. saline group). Mz.Cr at the doses of 50, 100 and 300 mg/kg reduced the castor oil-induced fluid accumulation to 108.30±0.47 (*P*<0.001 vs. castor oil group), 95.32±0.86 (*P*<0.001 vs. castor oil group) and 84.98±0.67 (*P*<0.001 vs. castor oil group) respectively. Atropine at the dose of 10 mg/kg decreased the intestinal fluid accumulation to 74.34±0.69 (*P*<0.001 vs. castor oil group) as shown in Figure 5.

***Effect of Mz.Cr on spontaneous and K*** ^+^ ***induced contractions***

**Figure 6:**
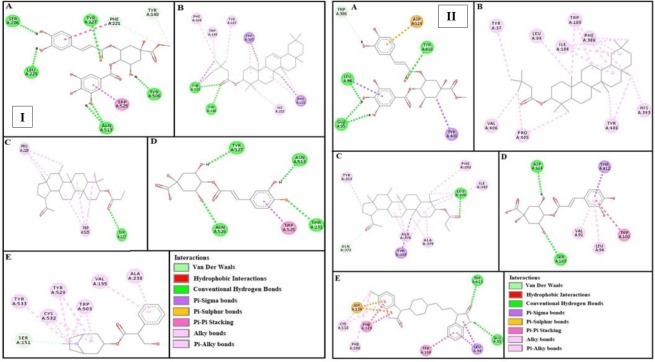
Panels [I] and [II] shows (A), (B), (C) and (D) interactions of methyl 4-O-galloylchlorogenate, β-amyrin-3-(3’-dimethyl) butyrate, lupeol-3-acetate and 4-caffeoylquinic acid against targets: musranic M3 and dopaminergic D2 receptors respectively. (E) represents atropine and domperidone interactions

Figure 6 shows comparative inhibitory effect of the plant extract, papaverine and verapamil against spontaneous and K^+ ^(80 mM)-induced contractions. Mz.Cr was found to be equally effective against spontaneous and K^+ ^(80 mM)-induced contractions with EC_50 _values of 0.11mg/ml (0.08-0.1, n=4) and 0.16 mg/ml (0.09-0.2, n=4) respectively as shown in Figure 6A. Papaverine also showed similar pattern of non-specific inhibitory response (Figure 6B) with respective EC_50 _values of 0.6 (0.3-1.3, n=4) and 0.4 μM (0.2-0.8, n=4), whereas, verapamil was found more potent against K^+ ^(80 mM)-induced contractions with EC_50 _value of 0.04 µM (0.03-0.06, n=4), as compared to spontaneous contractions (0.12 µM (0.10-0.20, n=3)) as shown in Figure 6C.

***Effect of Mz.Cr on ethanol-HCl induced ulcer***

**Figure 7:**
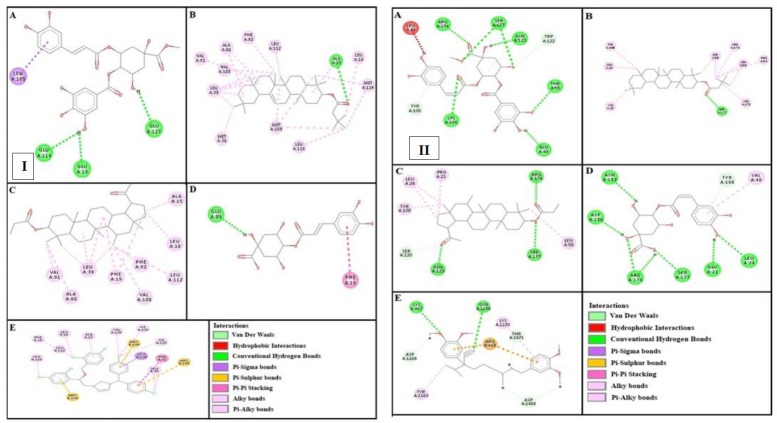
Panels [I] and [II] shows (A), (B), (C) and (D) interactions of methyl 4-O-galloylchlorogenate, β-amyrin-3-(3’-dimethyl) butyrate, lupeol-3-acetate and 4-caffeoylquinic acid against targets: calmodulin receptor and calcium channel respectively. (E) represents calmozolium and verapamil interactions

Mz.Cr in dose dependent manner (50-300 mg/kg) exhibited an anti-ulcer effect. Mz.Cr at 50, 100 and 300 mg/kg caused 21.1, 42.2 and 73.26% (*P*<0.001 vs. saline group) inhibition respectively. Omeprazole (20 mg/kg) exhibited 88.8% inhibitory effect (Table 2). Macroscopic observation showed the gastric mucosa of rats (Figure 7).

***Effect of Mz.Cr on charcoal meal transit time***

Mz.Cr hinders the charcoal meal to travel through the small intestine in a dose dependent manner. The distance travelled by the saline group was 82.29%. Mz.Cr at 50, 100 and 300 mg/kg dose shows inhibition of charcoal meal transit by 54.05, 51.57 and 47.25% respectively (*P*<0.001 vs. saline group). Atropine (0.1 mg/kg, IP) shows inhibitory effect of 44.23% (Table 3).

***Acute toxicity***

The extract did not show any mortality up to the dose of 5 g/kg.

***Docking evaluation***

**Figure 8:**
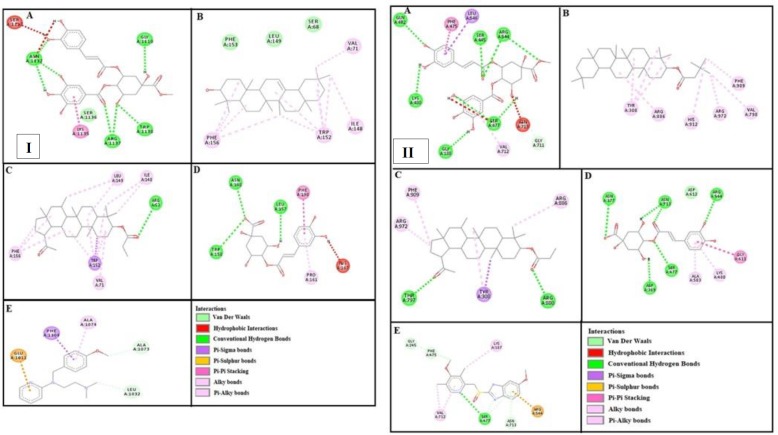
Panels [I] and [II] shows (A), (B), (C) and (D) interactions of methyl 4-O-galloylchlorogenate, β-amyrin-3-(3’-dimethyl) butyrate, lupeol-3-acetate and 4-caffeoylquinic acid against targets: histaminergic H_1_ receptor and H^+^/K^+^ ATPase respectively. (E) represents pyrilamine and omeprazole interactions

**Figure 9:**
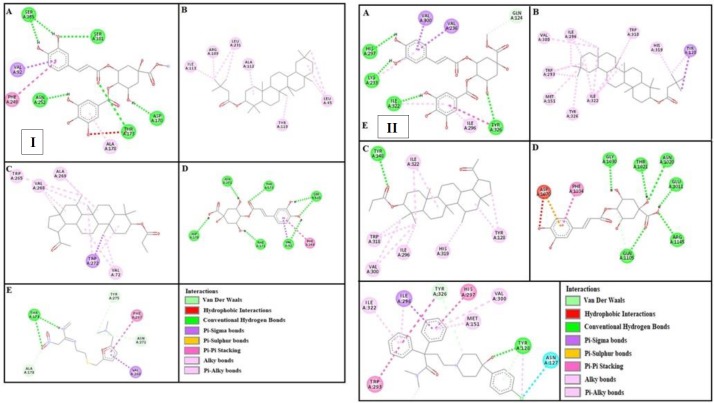
Panels [I] and [II] shows (A), (B), (C) and (D) interactions of methyl 4-O-galloylchlorogenate, β-amyrin-3-(3’-dimethyl) butyrate, lupeol-3-acetate and 4-caffeoylquinic acid against targets: histaminergic H_2_ and opioid mu receptors respectively. (E) represents ranitidine and loperamide interactions

**Figure 10:**
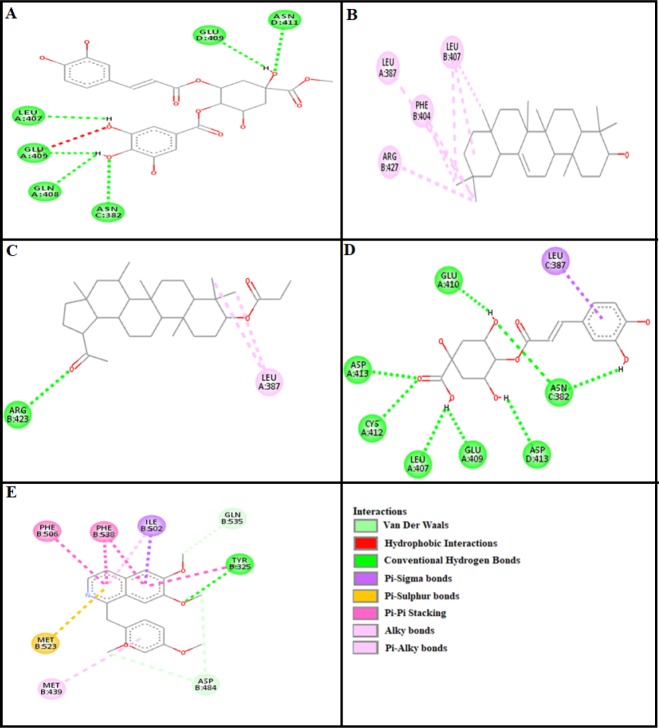
(A), (B), (C), (D) and (E) represents interactions of methyl 4-O-galloylchlorogenate, β-amyrin-3-(3’-dimethyl) butyrate, lupeol-3-acetate, 4-caffeoylquinic acid and papaverine against target: phosphodiesterase enzyme respectively

Assessment of E-value is an important contributor which helps in docking evaluation. Apart from this, other contributing factors include hydrogen bonding, pi-pi bonding and other hydrophobic interactions between ligand-protein complexes. Results of post dock analysis are given in Tables 4-6 and Table 7, showing number and binding residues of hydrogen bonds, pi-pi bonds and hydrophobic interactions respectively. Formation of bonding and interaction by β-amyrin-3-(3’-dimethyl) butyrate, methyl 4-O-galloylchlorogenate, 4-caffeoylquinic acid, lupeol-3-acetate and standard drugs against selected targets are shown in Figures 5-10 respectively.

## Discussion

Based on ethnopharmacological use of *M. zapota* in hyperactive gut diseases, such as colic and diarrhea, its extract was evaluated for the possible anti-diarrheal, anti-secretory, charcoal meal gastrointestinal motility and anti-ulcer effects in rodents. Isolated intestinal tissue was used for the elucidation of possible underlying mechanism(s) to rationalize aforementioned ethnomedicinal uses of the plant and it was further supported by virtual screening tool**s.**

Mz.Cr showed protective effect against castor oil induced diarrhea, similar to effect produced by loperamide, a standard drug (11). Castor oil induces diarrhea through its active metabolite i.e. ricinoleic acid. It is responsible for causing diarrhea through a series of actions including activation of small intestinal peristaltic activity with reduction of Na^+^-K^+^ATPase activity. These changes eventually result in disturbance in the intestinal mucosa, electrolyte permeability, hypersecretion of intestinal contents, and a slogging of the transport time in the intestine (20). Thus, a potential agent may exhibit its anti-diarrheal activity by these mechanisms. Intracellular Ca^2+ ^levels had a huge impact on secretary functions of the gastrointestinal organs which lead towards consequences such as discharge of gastric acids and intestinal fluid release. This effect might be affected by some drugs that hinder calcium influx (21). Mz.Cr shows protection against castor oil induced intestinal fluid secretions in mice. The anti-diarrheal and anti-secretory activities of Mz.Cr might be because of gastrointestinal relaxant component(s) present in the Mz.Cr.

Spontaneous contracting rabbit jejunum preparation is conventionally used to determine the spasmolytic impact, without the utilization of spasmogen (agonist). In jejunum, papaverine (Ca^2+^ influx and phosphodiesterase (PDE) inhibitor) and Mz.Cr both possess repressive effect on spontaneous as well as high K^+^-induced contractions with similar effect, where as verapamil, a specific calcium antagonist have inhibitory effect against the K^+^-induced contractions. Against spontaneous and K^+^-induced contractions Mz.Cr produces inhibitory pattern like papaverine does, which depicts that plant may be involved in dual mechanism(s) with CCB, in producing relaxation effect, like PDE enzyme(s) inhibition. PDE enzyme inhibitors augment the intracellular level of cyclic AMP which results in relaxation of smooth muscles (22). Traditionally *M. zapota* is used in colic and diarrhea, which is observed through its anti-diarrheal, anti-secretory, anti-ulcer and anti-spasmodic effects. This is expected as both Ca^2+ ^antagonists and PDE inhibitors possess an anti-diarrheal, anti-secretory and anti-spasmodic properties (15).

Various aggressive and protective factors play important role in acid release inside gastrointestinal tract. Any imbalance in these factors results in rupturing of mucosal protection and expose gastric lining to gastric acid leading to the sores called ulcers. To explore the anti-ulcer effect of Mz.Cr, ethanol-HCl induced gastric model was used which through variety of mechanisms stimulates ulcers including mucus exhaustion, mucosal damage, release of superoxide anion, hydroperoxide free radicals, all these mechanisms prolonged the tissue oxidative stress and release of inflammatory mediators (16). Marked inhibition on certain ethanol-HCl induced gastric lesions formation as compared to control group showed gastro protective effect of Mz.Cr. The potential of Mz.Cr to produce anti-ulcer effect might be due to its CCB effect, as Ca^2+^ antagonist are well known to demonstrate such effects (23). In pathophysiology of gastric ulcers, oxidative stress plays a vital role. Anti-oxidant and nitric oxide free radical scavenging activity has been reported by *M. zapota *(6), which may be responsible for its effectiveness as anti-ulcer agent.

In the small intestinal transit test, Mz.Cr produces suppression of the propulsion of charcoal marker at all test doses just like atropine sulphate a standard drug, that has been reported to have anticholinergic effect on intestinal transit (24). A decrease in the motility of gut muscles increases the stay of substances in the intestine, thus allows better water absorption. This finding suggests that Mz.Cr has the ability to influence the peristaltic movement of intestine thereby indicating the presence of an anti-motility activity. It is therefore presumed that the reduction in the intestinal propulsive movement in the charcoal meal model may be due to antispasmodic properties of the Mz.Cr (25).

The observed therapeutic effects of *M. zapota* may be due to the presence of phytochemicals, tannins and flavonoids, as these phytoconstituents are well known for gastrointestinal effects. Anti-diarrheal, anti-secretory, anti-ulcer and anti-spasmodic activities may be due to flavonoids. Beneficial role of tannins in diarrhea cannot be ignored (26). 

In acute toxicity testing, the Mz.Cr did not show any mortality up to the maximum dose (5 g/kg) tested, which shows the wide therapeutic range of *M. zapota.*

Molecular docking is an effective tool for evaluating the affinity of various protein targets that may possibly be associated with the pathophysiology of gastric disorders. The traditionally acclaimed use of *M. zapota *in the management of gastric related diseases has been supported with scientific evidence using virtual screening tool.

In this study, Auto Dock Vina program was used through PyRx (27). It uses gradient optimization method and it improves accuracy of binding mode predictions. Hydrogen bonding is reported to be significant in formation of ligand protein complex. In this study, we assessed affinity of ligands through E-value and number of hydrogen bonds against protein targets which imparts their influential effect in gastrointestinal diseases. Lower de-solvation energy is an indication of favorable ligand protein complex which is achieved with lower E-values (28). According to certain instances, no of pi-pi interactions formed by the ligand-target structural complex contributed to increase the stabilization of complex which is comparable to the stable interaction formed by H-bond. Other hydrophobic bonding likewise improves the partiality of ligand’s affinity for particular protein target (29). The affinity of ligands for respective targets was assessed on the basis of atomic energy value, hydrogen bonds, pi-pi interactions and hydrophobic bonding.

It has been found that 4-caffeoylquinic acid showed excellent score of binding against M_1_ receptor with lowest E-value. This binding efficacy is greater than majority of the target proteins with better affinity as compared to the other test compounds and standard drugs. Thus, this result suggests that it showed maximum affinity for binding with M_1_ receptor. Order of affinity of the test compounds for M_1_ receptor was; 4-caffeoylquinic acid>pirenzepine>methyl 4-O-galloylchlorogenate>β-amyrin-3-(3’-dimethyl) butyrate>lupeol-3-acetate. Order of affinity of the test compounds for adrenergic α_1 _receptor was found to be; phenoxy benzamine>4-caffeoylquinic acid>methyl 4-O-galloylchlorogenate>lupeol-3-acetate>β-amyrin-3-(3’-dimethyl) butyrate. Compounds with higher affinity all together formed stronger pi–pi bonds, high number of hydrophobic interactions and polar hydrogen bonding against M_1_ and α_1_ receptors, piranzapine showed only π–π interaction while phenoxy benzamine showed H-bonding along with π–π interactions as well. The order of affinity for ligands against M_3_ receptor was found as; 4-caffeoylquinic acid>atropine>methyl 4-O-galloylchlorogenate>β-amyrin-3-(3’-dimethyl) butyrate>lupeol-3-acetate. Order of affinity of the test compounds for dopaminergic D_2_ receptor was found as; 4-caffeoylquinic acid>methyl 4-O-galloylchlorogenate>lupeol-3-acetate>β-amyrin-3-(3’-dimethyl) butyrate>domperidone. Alongside hydrogen and hydrophobic interactions, different types of interactions, for example alkyl, pi-alkyl and vander waal interactions are appeared with high proclivity by test compounds. Amino acids; TYR 408, LEU 94, TRP 413 and ASP 114 are found to be important. Methyl 4-O-galloylchlorogenate, 4-caffeoylquinic acid and domperidone exhibited bonding with ASP 114, a stable amino acid residue (30). The affinity order of ligands against calmodulin was found as; 4-caffeoylquinic acid>methyl 4-O-galloylchlorogenate>calmidazolium> lupeol-3-acetate>β-amyrin-3-(3’-dimethyl) butyrate. In addition, hydrogen bond is considered to be vital for complex of ligand with calmodulin. The affinity order for test compounds for voltage gated L-Type calcium channel was found as; methyl 4-O-galloylchlorogenate>4-caffeoylquinic acid>verapamil>lupeol-3-acetate>β-amyrin-3-(3’-dimethyl) butyrate. Methyl 4-O-galloylchlorogenate, 4-caffeoylquinic acid and lupeol-3-acetate showed interactions with ARG569 which helps in making non-covalent bonds (salt bridge) (31). Order of affinity of test compounds for histaminergic H_1_ receptor was found to be: piranzapine>methyl 4-O-galloylchlorogenate> 4-caffeoylquinic acid>lupeol-3-acetate>β-amyrin-3-(3’-dimethyl) butyrate. Ligands are not engaged with making any solid interactions on stated restricting sites. Order of affinity of test compounds for H^+^/K^+ ^ATPase receptor was found as; omeprazole>4-caffeoylquinic acid>β-amyrin-3-(3’-dimethyl) butyrate>lupeol-3-acetate >methyl 4-O-galloylchlorogenate. Hydrogen and hydrophobic associations are observed to be essential but no such interactions of test compounds with stated restricting site were seen. In this regard, SER 477 is considered as important and vital amino acid. The affinity order of ligands against histaminergic H_2_ receptor was found as; ranitidine>4-caffeoylquinic acid>β-amyrin-3-(3’-dimethyl) butyrate>lupeol-3-acetate>methyl 4-O-galloylchlorogenate. Order of affinity of the test compounds for mu-opioid receptor was found as: 4-caffeoylquinic acid>methyl 4-O-galloylchlorogenate> loperamide>lupeol-3-acetate>β-amyrin-3-(3’-dimethyl) butyrate. Order of affinity of test compounds for phosphodiesterase enzyme was found as: papaverine >methyl 4-O-galloylchlorogenate>4-caffeoylquinic acid >lupeol-3-acetate>β-amyrin-3-(3’-dimethyl) butyrate. Ligands having high restricting proclivity shaped interacts with TYR272 and VAL270.

It is revealed that 4-caffeoylquinic acid and methyl 4-O-galloylchlorogenate showed more affinity than lupeol-3-acetate and β-amyrin-3-(3’-dimethyl) butyrate. Hydrophobic interactions were shown by ligands with high affinity. Essential amino acids of arginine family are important in the binding site which is involved in interactions with all these ligands (32). 

**Table 1:** Effect of the *Manilkara zapota* crude extract (Mz.Cr) and loperamide against castor oil-induced diarrhea in mice

**Treatment** **(mg/kg)**	**No of mice (out of 5) with diarrhea**	**Protection** **(%)**
Saline (10 mL/kg) + castor oil	5	0
Mz.Cr (50 mg/kg) + castor oil	4	20
Mz.Cr (100 mg/kg) + castor oil	3	40
Mz.Cr (300 mg/kg) + castor oil	1^*^	80
Loperamide (10 mg/kg) + castor oil	0^**^	100

*P<0.05, **P<0.01 compared to saline group, data analyzed by Chi-squared test

**Table 2:** Protective effect of *Manilkara zapota* crude extract (Mz.Cr) and omeprazole against ethanol-HCl induced gastric ulcers in rats

**Treatment**	**Ulcer Index **	** % Inhibition**
Saline 10 mL/kg + Ethanol-HCl	9.0 ± 0.07	-
Mz.Cr (50 mg/kg) + Ethanol-HCl	7.1 ± 0.20^***^	21.1
Mz.Cr (100 mg/kg) + Ethanol-HCl	5.2 ± 0.14^***^	42.2
Mz.Cr (300 mg/kg) + Ethanol-HCl	2.4 ± 0.14^***^	73.26
Omeprazole (20 mg/kg) + Ethanol-HCl	1 ± 0.11^***^	88.8

***P<0.001 compared to control saline group, one-way analysis of variance, followed by *Post hoc* Tukey’s test, n=5

**Table 3:** Effect of *Manilkara zapota* crude extract (Mz.Cr) and atropine on charcoal meal transit time in rats

**Treatment (mg/kg)**	**Mean length of Intestine (cm)**	**Distance Moved by Charcoal (cm)**	**Intestinal transit %**
Saline(10 mL/kg)	86.66 ± 0.6	71.32 ± 0.6	82.29
Mz.Cr (50 mg/kg)	86.32 ± 0.3	46.66 ± 0.4^***^	54.05
Mz.Cr (100 mg/kg)	85.32 ± 0.3	44.00 ± 0.5^***^	51.57
Mz.Cr (300 mg/kg)	84.99 ± 0.4	40.32 ± 0.6^***^	47.25
Atropine (0.1 mg/kg, i.p.)	86.66 ± 0.4	39.66 ± 0.4^***^	44.23

***P<0.001 compared to control saline group, one-way analysis of variance followed by *Post hoc* Tukey’s test, n=5

**Table 4:** E-values (Kcal/mol) of best docked poses of methyl 4-O-galloylchlorogenate, β-amyrin-3-(3’-dimethyl) butyrate, lupeol-3-acetate, 4-caffeoylquinic acid and standard drugs against targets: adrenergic α_1_ receptor, muscranic M_1_, muscranic M_3_, dopaminergic D_2_, calmodulin, mu-opioid, voltage gated L-Type calcium channel, histaminergic H_1_, histamergic H_2_, H^+^/K^+^ ATPase pump and phosphodiesterase enzyme

**Target Proteins**	**PDB ID**	**β-amyrin-3-(3' dimethyl) butyrate**	**4-Caffeoylquinic acid**	**Methyl 4-O-galloylchlorogenate**	**Lupeol-3-acetate**	**Standard drugs**
Adrenergic α_1_	3538	-10.3	-8.4	-8.5	-9.9	-8.0^A^
Muscranic M_1_	5CXV	-10.0	-7.6	-9.1	-10.9	-9.0^B^
Muscranic M_3_	4U14	-9.3	-7.8	-8.9	-9.5	-8.6^C^
Dopaminergic D_2_	6CM4	-9.7	-8.4	-9.4	-9.5	-10.6^D^
Calmodulin	1CTR	-8.9	-6.3	-7.1	-8.4	-8.3^E^
Calcium channel	1T3S	-9.3	-7.4	-7.4	-8.9	-7.9^F^
Histaminergic H_1_	3RZE	-8.5	-7.1	-6.9	-8.0	-5.7^G^
H^+^/K^+^ ATPase	5YLU	-9.7	-9.2	-10.9	-10.3	-8.4^H^
Histaminergic H_2_	P25021	-8.7	-8.6	-9.7	-8.8	-6.1^I^
Mu-opioid	5C1M	-10.5	-7.3	-8.4	-9.4	-9.2^J^
Phosphodiesterase enzyme	3G4K	-10.5	-9.1	-8.8	-9.7	-8.3^K^

Standard inhibitors or activator of pathways are: (A) phenoxy benzamine, (B) pirenzapine, (C) atropine, (D) domperidone, (E) calmozolium, (F) verapamil, (G) pyrilamine,(H) omeprazole, (I) ranitidine, (J) loperamide and (K) papaverine

**Table 5:** Hydrogen bonds (H-bonds) formed by methyl 4-O-galloylchlorogenate, β-amyrin-3-(3’-dimethyl) butyrate, lupeol-3-acetate, 4-caffeoylquinic acid and standard drugs against targets: adrenergic α_1_ receptor, muscranic M_1_, muscranic M_3_, dopaminergic D_2_, calmodulin, mu-opioid, voltage gated L-Type calcium channel, histaminergic H_1_, histamergic H_2_, H^+^/K^+^ ATPase pump and phosphodiesterase enzyme

**Target Proteins**	**PDB ID**	**β-amyrin-3-(3' dimethyl) butyrate**		**4-caffeoylquinic acid**		**Methyl 4-O-galloylchlorogenate**		**Lupeol-3-acetate**		**Standard drugs**	
		**H-bonds**	**Amino Acids**	**H-bonds**	**Amino Acids**	**H-bonds**	**Amino Acids**	**H-bonds**	**Amino Acids**	**H-bonds**	**Amino Acids**
Adrenergic α_1_	35348	0	-	6	SER 302(2) THR 181 SER 180	6	LYS 271 SER 251 PRO 229 ARG 206 GLN 210(2)	0	-	0^A^	-
Muscranic M_1_	5CXV	0	-	5	TYR 381 TYR 106 THR 189 ASN 382	6	TYR 82 TYR 381 SER 388 ILE 180 LEU 183 THR 189	0	-	2^B^	ILE 180 TYR 381
Muscranic M_3_	4U14	2	TYR 529 TYR 148	4	TYR 127 ASN 513 ASN 526 THR 231	6	SER 226 TYR 127 TYR 506 LEU 225 ASN 513 ASN 513	1	TYR127	0^C^	-
Dopaminergic D_2_	6CM4	0	-	2	ASP 114 SER 193	4	GLU 95 GLU 95 LEU 94 TYR 416	1	LEU 206	2^D^	TYR 413 GLU 95
Calmodulin	1CTR	1	ALA 15	1	GLU 84	3	GLU 114 GLU 14 GLU 127	0	-	0^E^	-
Calcium channel	1T3S	1	SER 177	7	ASN 123 ASP 126 ARG 174 ARG 174 SER 177 GLU 22 LEU 24	7	ARG 174 SER 177 SER 177 ASN 123 LYS 104 THR 55 GLU 49	3	ARG 174 SER 177 ASN 123	2^F^	GLN 1156 ILE 381
Histaminergic H_1_	3RZE 3RZE	0	-	3	ASN 198 LEU 157 TRP 158	4	ASN 1132 GLY 1110 ARG 1137 TRP 1138	1	ARG 53	**0** ^G^	-
H^+^/K^+^ ATPase	5YLU	0	-	5	ASN 377 ASN 713 ARG 544 SER 477 ASP 369	6	GLN 482 LYS 480 SER 445 ARG 544 GLY 188 SER 477	2	THR 529 ARG 880	1^H^	SER 477
Histaminergic H_2_	P25021	0	-	6	ASN 271 THR 173 ASP 170 PHE 171 VAL 92 SER 185	5	SER 185 SER 181 ASN 252 THR 173 ASP 170	0	-	2^I^	THR 173
Mu-opioid	5C1M	0	-	6	GLY 1030 THR 1021 ASN 1020 GLU 1011 ARG 1145 GLN 1105	4	HIS 287 LYS 233 ILE 322 TYR 326	1	TYR 148	1^J^	TYR 128
Phosphodiesterase enzyme	3G4K	0	-	7	GLU 410 ASN 382 ASP 413 GLU 409 LEU 407 CYS 412 ASP 413	6	LEU 407 GLU 409 GLN 408 ASN 382 GLU 409 ASN 411	1	ARG 423	1^K^	TYR 325

Standard inhibitors or activators are: (A) phenoxy benzamine, (B) pirenzapine, (C) atropine, (D) domperidone, (E) calmidazolium, (F) verapamil (G)omeprazole, (I) ranitidine, (J) loperamide and (K) papaverine. Amino acids are: ALA, alanine; ARG, arginine; ASN, asparagine; ASP, aspartic acid; CYS, cysteine; GLN, glutamine; GLU, glutamic acid; GLY, glycine; HIS, histidine; ILE, isoleucine; LYS, lysine; MET, methionine; PHE, phenylalanine; PRO, proline; SER, serine; THR, threonine; TRP, tryptophan; TYR, tyrosine and VAL, valine

**Table 6:** Pi-Pi bonds (p-p bonds) formed by methyl 4-O-galloylchlorogenate, β-amyrin-3-(3’-dimethyl) butyrate, lupeol-3-acetate, 4-caffeoylquinic acid and standard drugs against targets: adrenergic α_1_ receptor, muscranic M_1_, muscranic M_3_, dopaminergic D_2_, calmodulin, mu-opioid, voltage gated L-Type calcium channel, histaminergic H_1_, histaminergic H_2_, H^+^/K^+^ ATPase pump and phosphodiesterase enzyme

**Proteins**	**PDB ID**	**β-amyrin-3-(3'-dimethyl) butyrate**		**4-caffeoylquinic acid**		**methyl 4-O-galloylchlorogenate**		**Lupeol-3-acetate**		**Standard drugs**	
		**π-πbonds**	**Amino Acids**	**π-πbonds**	**Amino Acids**	**π-πbonds**	**Amino Acids**	**π-πbonds**	**Amino Acids**	**π-πbonds**	**Amino Acids**
Adrenergic α_1_	35348	0	-	0	-	1	LEU 208	1	TYR 186	3^A^	PHE 299 TRP 295 PHE 298
Muscranic M_1_	5CXV	0	-	0	-	1	TRP 400	2	TYR 404 TRP 400	1^B^	TYR 404
Muscranic M_3_	4U14	2	TYR 529 TRP 525	1	TRP 525	2	TRP 525 PHE 221	0	-	0^C^	-
Dopaminergic D_2_	6CM4	0	-	2	THR 412 TRP 100	3	ASP14 TYR 408 LEU 94	1	TYR 209	4^D^	ASP 114 PHE 389 LEU 94 TRP 100
Calmodulin	1CTR	0	-	1	PHE 19	1	LEU 105	0	-	5^E^	MET 144 MET 145 MET 109 LEU 105 PHE 92
Calcium channel	1T3S	0	-	0	-	0	-	0	-	1^F^	ARG 413
Histaminergic H_1_	3RZE	0	-	1	PHE 190	1	LYS 1135	1	TRP 152	2^G^	PHE 1104 GLU 1011
H^+^/K^+^ ATPase	5YLU	0	-	1	GLY 611	2	LEU 546 PHE 475	1	TYR 308	1^H^	ARG 544
Histaminergic H_2_	P25021	0	-	2	PHE 249 VAL 92	2	VAL 92 PHE 249	1	TRP 272	2^I^	PHE 267 VAL 268
Mu-opioid	5C1M	1	TYR 128	1	PHE 1104	2	VAL 236 VAL300	0	-	3^J^	ILE 296 HIS 297 TRP 293
Phosphodiesterase enzyme	3G4K	0	-	1	LEU 387	0	-	0	-	4^K^	PHE 506 PHE 538 ILE 502 MET 523

Standard inhibitors or activatorsare: (A) phenoxy benzamine, (B) piranzapine, (C) atropine, (D) domperidone, (E) calmozolium, (F) verapamil, (G) pyrilimine,(H) omeprazole, (I) ranitidine, (J) loperamide and (K) papaverine. Amino acids are: ALA, alanine; GLN, glutamine; GLY, glycine; HIS, histidine; LYS, lysine; PHE, phenylalanine; SER, Serine; TRP, tryptophan and TYR, tyrosine

**Table 7:** Hydrophobic interactions formed by methyl 4-O-galloylchlorogenate, β-amyrin-3-(3’-dimethyl) butyrate, lupeol-3-acetate, 4-caffeoylquinic acid and standard drugs against targets: adrenergic α_1_ receptor, muscranic M_1_, muscranic M_3_, dopaminergic D_2_, calmodulin, mu-opioid, voltage gated L-Type calcium channel, histaminergic H_1_, histamergic H_2_, H^+^/K^+^ ATPase pump and phosphodiesteraseenzyme

**Target proteins**	**PDB ID**	**β-amyrin-3-(3'-dimethyl) butyrate**	**4-caffeoylquinic acid**	**methyl 4-O-galloylchlorogenate**	**Lupeol-3-acetate**	**Standard drugs**
Adrenergic α_1_	35348	ILE 300(2), 307, 193 TYR 186(2), 196 LEU 190(2), 296(2), 303(2), 288	ILE 98	TYR 254 ALA 274	ILE 193(2), 292(2) LEU 303(2), 190(2), 296(2)	ALA 184 ILE 98 CYS 101 VAL 169 VAL 168^A^
Muscranic M_1_	5CXV	ARG 123, LEU 367, 64, 56 ALA 363, 424, ILE 119 LYS 423	ALA 196	ILE 180 LEU 183	TYR 381,82 PRO 186 VAL 385	LEU 183 TYR 106^B^
Muscranic M_3_	4U14	TYR 127, TRP 143 PHE 124, ILE 222	-	PHE 221 TYR 248	PRO 228(3) TRP 525(3)	TYR 533 TYR 529 CYS 532 TRP 503 VAL 155 ALA 238^C^
Dopaminergic D_2_	6CM4	TYR 37, 408 LEU 94, TRP 100 ILE 184, PHE 386 HIS 393, PRO 405 VAL 406	VAL 91 LEU 94	ASP 114 TRP 386	TYR 213 PHE 202 ILE 383 ALA 376,379 GLN 373	PHE 202 ILE 383 TYR 213 ALA 376 GLN 373^D^
Calmodulin	1CTR	VAL 91, 108 ALA 88, PHE 92 LEU 112, 39, 116, 18 MET 36, 109, 124	PHE 19	-	VAL 91,108 ALA 88,15 LEU 39,112.18 PHE 19,92	PHE 19 LEU 116 LEU 18 ALA 15 VAL 136 ALA 100^E^
Calcium channel	1T3S	TYR 108 LEU 24, 58, 59, 175 VAL 23, ARG 174 PHE 62	TYR 108 VAL 48	TRP 122 TYR 108	LEU 24, 58 PRO 21 TYR 108 SER 120	LYS 1170 THR 1471 ASP 1468 TYR 1163^F^
Histaminergic H_1_	3RZE	VAL 71 ILE 148 TRP 152 PHE 156	PRO 161	SER 1136	ILE 148 LEU 149 VAL 71	ALA 1074 ALA 1073 LEU 1032^G^
H^+^/K^+^ ATPase	5YLU	TYR 308 ARG 886, 972 HIS 912, VAL 798 PHE 909	ASP 612 ALA 503 LYS 480 GLY 611	GLY 711 VAL 712	ARG 972, 886 PHE 909	GLY 245 PHE 475 VAL 712 ASN 713 LYS 187^H^
Histaminergic H_2_	P25021	ILE 113 ARG 109 LEU 231, 45 ALA 112 TYR 119	-	PHE 249 ALA 178	ALA 269 VAL 72, 268 TRP 265	TYR 275 ASN 271 ALA 178^I^
Mu-opioid	5C1M	VAL 300 ILE 296, 322 TRP 293, 318 MET 151 TYR 326 HIS 319	ASP 1070 GLN 1105	ILE 296 ILE 322 GLN 124	ILE 322, 296 TRP 318 VAL 300 HIS 319 TYR 128	ILE 296 TYR 326 VAL 300 MET 151 ILE 322^J^
Phosphodiesterase enzyme	3G4K	LEU 426, 407, 387 PHE 415 ARG 423 ASP 413	LYS 480 ALA 503	VAL 712 GLY 711	LEU 387(2)	GLN 535 ASP 484 MET 439^K^

Standard inhibitors or activators are: (A) phenoxy benzamine, (B) pirenzepine, (C) atropine, (D) domperidone, (E) calmidazolium, (F) verapamil, (G)pyrilamine, (H) omeprazole ,(I) ranitidine , (J) loperamide and (K) papaverine.Amino acids are: ALA, alanine; ARG, arginine; ASN, asparagine; ASP, aspartic acid; CYS, cysteine; GLN, glutamine; GLU, glutamic acid; GLY, glycine; HIS, histidine; ILE, isoleucine; LYS, lysine; MET, methionine; PHE, phenylalanine; PRO, proline; SER, serine; THR, threonine; TRP, tryptophan; TYR, tyrosine and VAL, valine

## Conclusion

*M. zapota* exhibited anti-diarrheal, anti-secretary, anti-spasmodic, anti-motility and anti-ulcer effects. The plant constituents: caffeoylquinic acid and methyl 4-O-galloylchlorogenate showed high binding affinities (E-value≥-6.5 Kcal/mol) against histaminergic H_2_ receptors, H^+^/K^+^ ATPase pump and voltage gated L-type calcium channels, while showed moderate affinities (E-value≥8 Kcal/mol) against histaminergic H_1_, muscarinic M_1_, muscarinic M_3_, mu-opioid, whereas revealed lower affinities (E-value≥9.5 Kcal/mol) vs. calmodulin, adrenergic α_1_, phosphodiesterase enzyme and dopaminergic D_2_ receptors. Lupeol-3-acetate and β-amyrin-3-(3’-dimethyl) butyrate exhibited weak affinities against aforementioned targets.
